# Supplementary material for: Overcoming low initial coulombic efficiencies of Si anodes through prelithiation in all-solid-state batteries
Source: Nat Commun. 2024 Apr 6;15:2991. doi: 10.1038/s41467-024-47352-y (PMC10998844; doi:10.1038/s41467-024-47352-y)
Supplement: Supplementary file 1 — Supplementary Information [file 41467_2024_47352_MOESM1_ESM.pdf]

Supplementary Information

Overcoming Low Initial Coulombic Efficiencies of Si Anodes Through Prelithiation in All-solid-state Batteries

So-Yeon Ham[1], Elias Sebti[2], Ashley Cronk[1], Tyler Pennebaker[2], Grayson Deysher[1], Yu-Ting Chen[1], Jin An Sam Oh[3], Jeong Beom Lee[4], Min Sang Song[4], Phillip Ridley[5], Darren H. S. Tan[5], Raphaële J. Clément[2], Jihyun Jang[5,6,*], and Ying Shirley Meng[5,7,*]

[1]Materials Science and Engineering Program, University of California San Diego, La Jolla, 92093, United States.
[2]Materials Department and Materials Research Laboratory, University of California, Santa Barbara, California 93106, United States.
*[3]Insitute of Materials, Research, and Engineering, Agency of Science, Technology, and Research (A*STAR), Singapore*
[4]LG Energy Solution. Ltd., LG Science Park, Magokjungang 10-ro, Gangseo-gu, Seoul 07796, Korea.
[5]Department of NanoEngineering, University of California San Diego, La Jolla, California 92093, United States.
[6]Department of Chemistry, Sogang University, Seoul, 04107, Republic of Korea
[7]Pritzker School of Molecular Engineering, University of Chicago, Chicago, Illinois 60637, United States.
**Correspondence to: jihyunjang@sogang.ac.kr, shirleymeng@uchicago.edu*

Li_1Si Pressing Condition	Relative Li mol.% (not T2 adjusted)	Relative Li-Si mol.% (not T2 adjusted)	Li T2 (stretched exp) [s]	Li-Si T2 (stretched exp) [s]	Normalized Relative Li mol.% (T2 adjusted)	Nomalized Relative Li-Si mol.% (T2 adjusted)
0 MPa, 0 s	**75.8**	**24.2**	0.000467	N/A	N/A	N/A
100 MPa, 30 s	7.7	92.3	0.000467	7.10E-05	**6.9**	**93.1**
200 MPa, 30 s	7.6	92.4	0.000467	1.08E-04	**7.1**	**92.9**
400 MPa, 30 s	3.4	96.6	0.000467	6.80E-05	**3.0**	**97.0**
200 MPa, 3 min	2.0	98.0	0.000467	6.90E-05	**1.8**	**98.2**

Table S1. Relative ratio (in Li mol.%) of observed ^{7}Li ssNMR signal intensity corresponding to metallic Li and to a Li-Si alloy. ^{7}Li ssNMR spectra were acquired on vortex-mixed powders with nominal composition Li_1Si after application of pressures varying from 0 to 400 MPa for 30 s to 3 minutes. The relative ratios are given before and after adjusting for spin-spin (T_2*) relaxation of the ^{7}Li ssNMR signal during data acquisition. The T_2* relaxation time of Li metal was measured on a pure SLMP sample, fitted to a single stretched exponential decay function, and used to scale the metallic Li signal observed in the spectra obtained on all samples. The T_2* relaxation time for the diamagnetic components, including the Li-Si alloy phase, was measured on each sample due to expected changes in Li-Si alloy composition with pressure, and fitted to a stretched exponential. For the unpressed sample, the T_2* of the Li-Si signal could not be determined as it evolved during the T_2* measurement (see **Figure S3**).

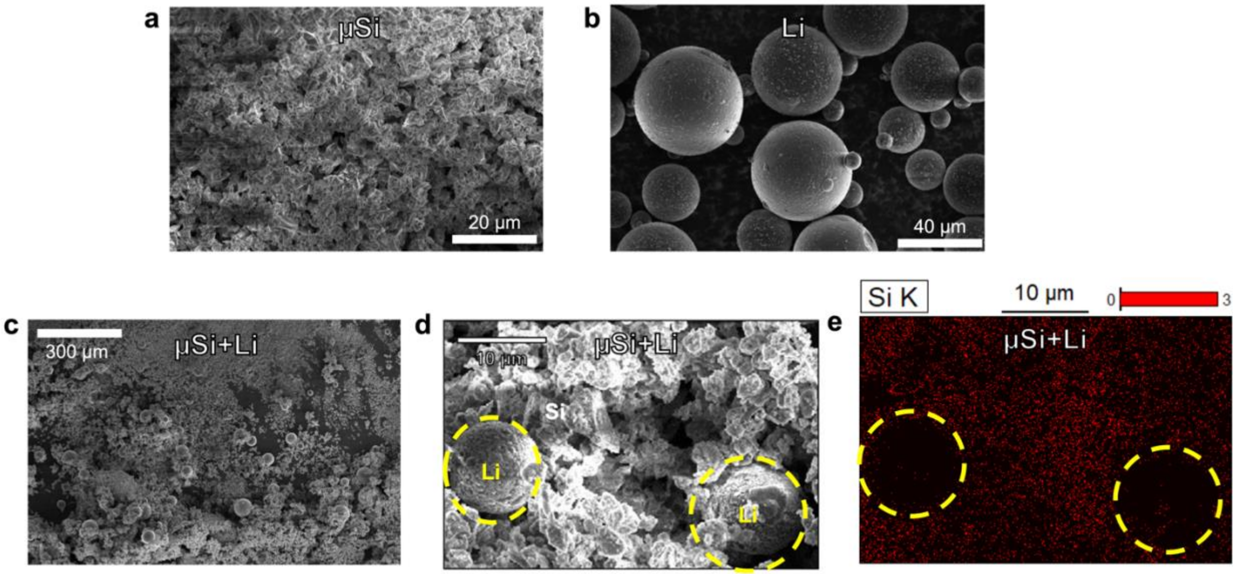

Figure S1. Morphology of μSi and SLMP. SEM images of (a) μSi, (b) SLMP (Li), (c) vortex mixed μSi and Li. (d) SEM image of vortex mixed μSi and Li and corresponding Si EDS from the same area.

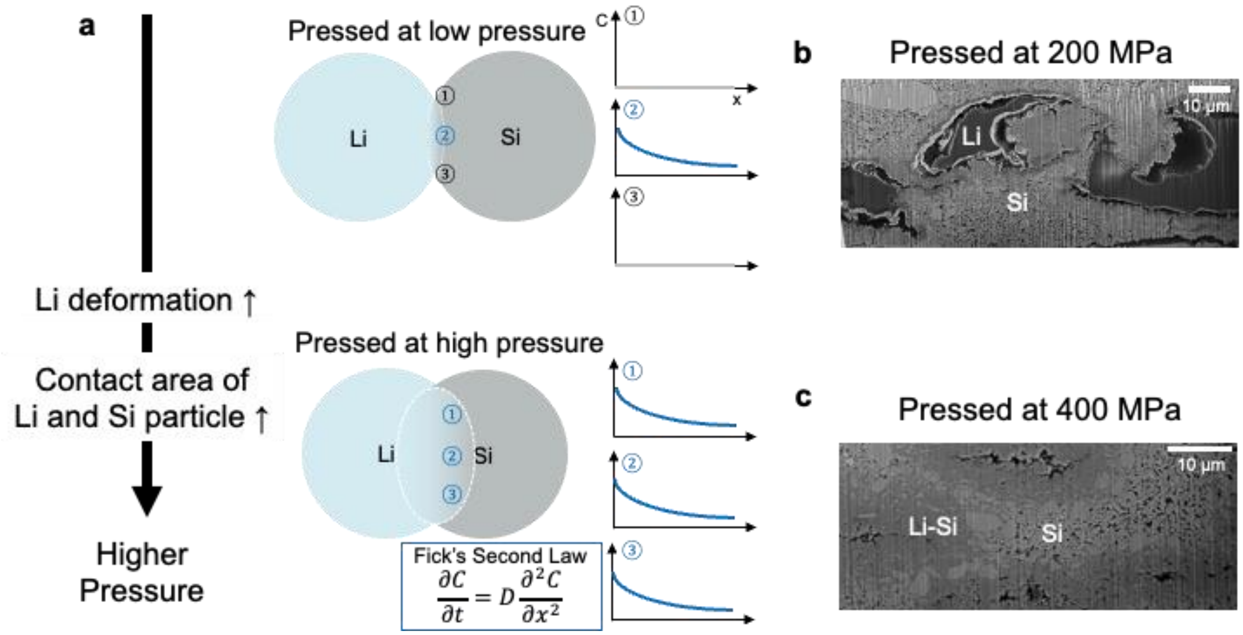

Figure S2. Schematics and morphology of Li_1Si pressed at low and high pressure. (a) Schematic of pressure-induced lithiation at low pressure (top) and high pressure (bottom). The hypothetical concentration of Li with respect to distance from the Li and Si contact point is shown on the right side. (b) Cross-sectional FIB/SEM image of Li_1Si at 200 MPa (c) Cross-sectional FIB/SEM image of Li1Si at 400 MPa.

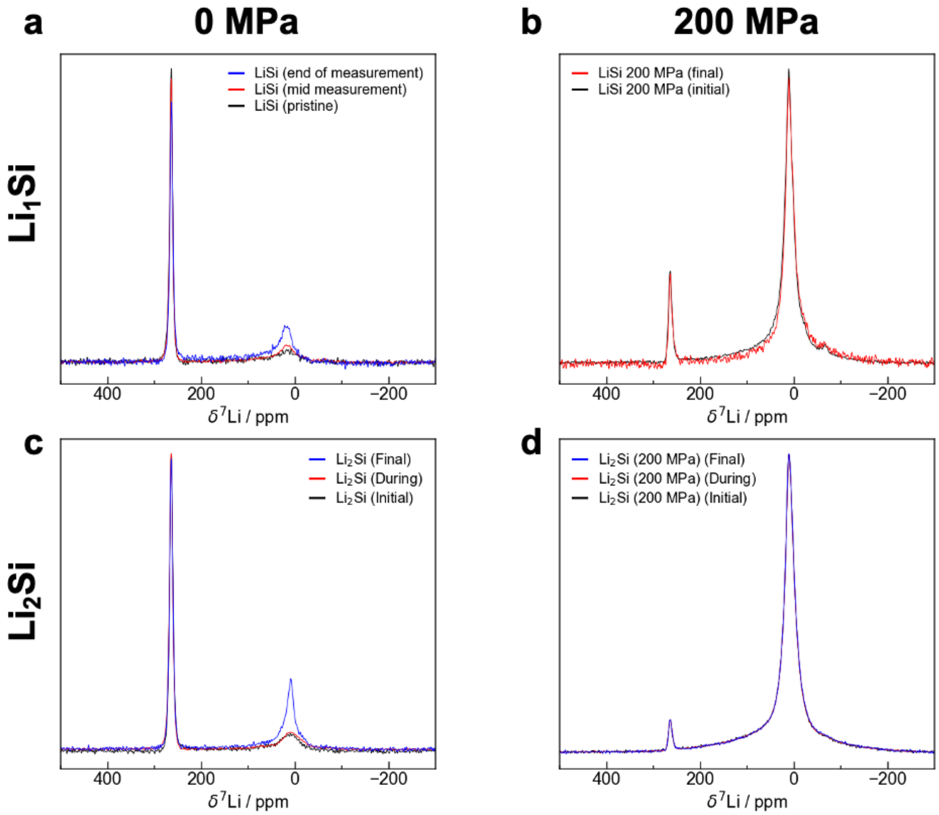

Figure S3. ^{7}Li ssNMR spectra of vortex-mixed Li_xSi. ^{7}Li ssNMR spectra of two compositions of (a, b) Li_1Si and (c, d) Li_2Si. The samples were either unpressed (a, c) or pressed at 200 MPa for 30 s. For each sample, ^{7}Li ssNMR spectra were obtained before, during, and after the spin-spin (T_2*) relaxation time measurement. For the two unpressurized samples (a, c), the Li-Si alloy signal grows during the T_2* measurement, precluding an accurate estimation of its T_2* relaxation time.

***Homogeneity of NMR* spectra**

We conducted additional fits on each of the spectra presented in **Figure 2c** of the manuscript. Fits are presented in **Figure S4** below. The Li-Si resonance for the unpressed sample is centered at 16.5 ppm, which corresponds well to the reported resonance for Li_7Si_{31}. The spectrum obtained on the sample pressed at 100 MPa for 30 s exhibits resonances at 69.4 ppm and 3 ppm, which are tentatively assigned to $Li_{21}Si_5$ and $Li_{15}Si_4$, respectively.[1] Fits for the spectra obtained on the samples pressed at 200 MPa and 400 MPa for 30 s as well as 200 MPa for 3 min are nearly identical and are fit with a single sharp resonance centered between 10.2-11.5 ppm, and a much broader resonance centered between 15-30 ppm. The sharp resonance in these samples is tentatively assigned to $Li_{13}Si_4$, while the broad component is due to a combination of other Li_xSi_y phases present.[1] An additional, weak signal at –67 ppm is observed in each spectrum and could not be assigned to a known Li-Si phase.

Based on the above results, it appears that the least homogenous sample is the sample pressed at 100 MPa for 30 s as it likely contains both $Li_{21}Si_5$ and $Li_{15}Si_4$. While the unpressed sample spontaneously formed some Li_7Si_3, it is still mostly composed of unincorporated Li metal. Pressing the Li-Si mixtures at higher pressures and for longer durations forms a larger phase fraction of the Li-Si alloy and appears to yield a more consistent mixture of Li_xSi_y phases, mostly consisting of $Li_{13}Si_4$. While consistency across samples should not be conflated with a homogenous distribution of phases throughout individual samples (NMR does not provide any information on the spatial distribution of the phases), the larger pressure applied on these samples likely forces more intimate contact and reactions between the SLMP and Si alloy that drives the formation of mostly $Li_{13}Si_4$.

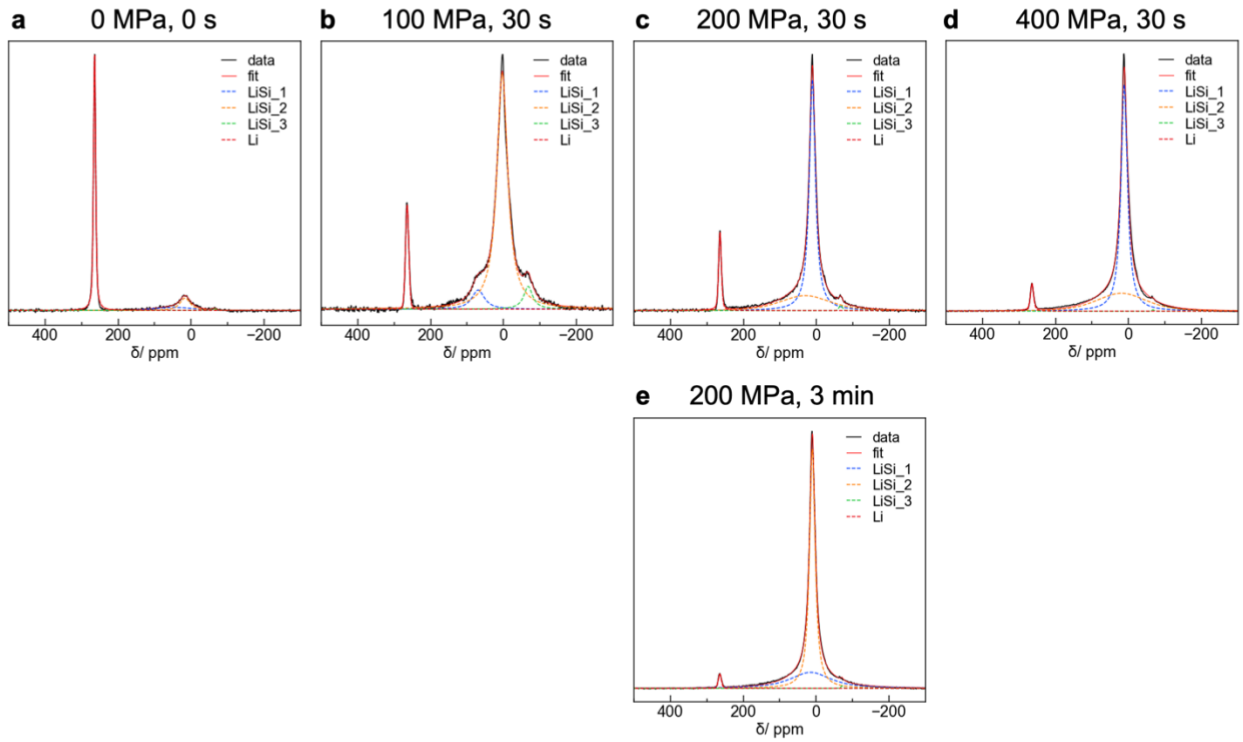

Figure S4. **Fits conducted on ^{7}Li ssNMR spectra obtained on Li_1Si mixtures pressed under various conditions.** (a) pristine without pressure. Li_1Si pressed (b) at 100 MPa for 30 s (c) at 200 MPa for 30 s (d) at 400 MPa for 30 s (e) at 200 MPa for 3 min. All spectra were obtained at 18.8 T with a spin-echo pulse sequence using 30° and 60° flip angles under static conditions.

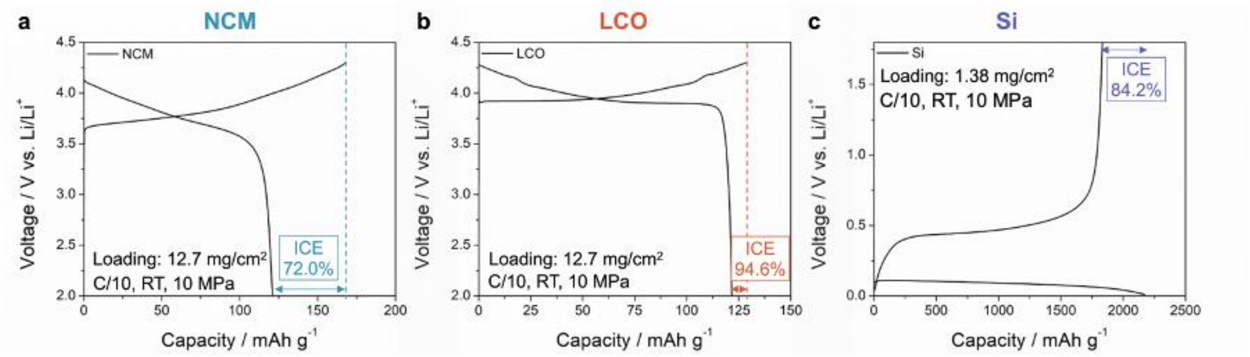

Figure S5. First cycle performance of half-cells. Half-cell data of (a) NCM, (b) LCO, and (c) Si with Li metal counter electrode. All cells were cycled at C/10, room temperature, and 10 MPa.

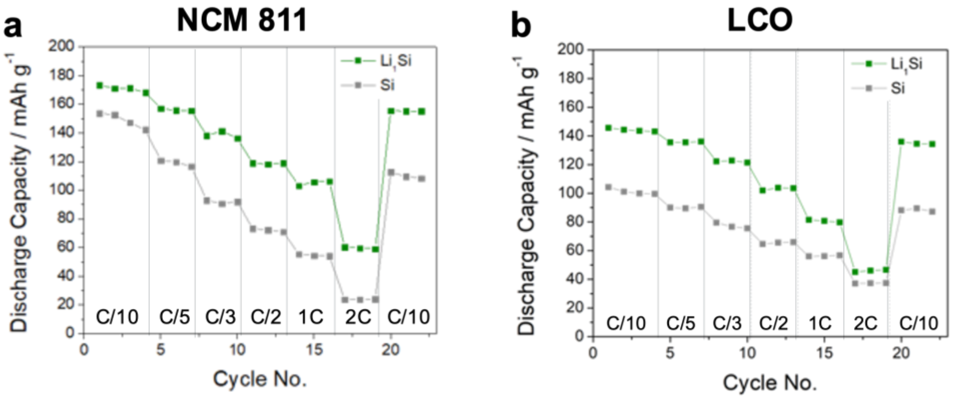

Figure S6. Rate performance of Si and Li$_1$Si. Rate tests of (a) NCM 811 and (b) LCO paired with Si and Li$_1$Si

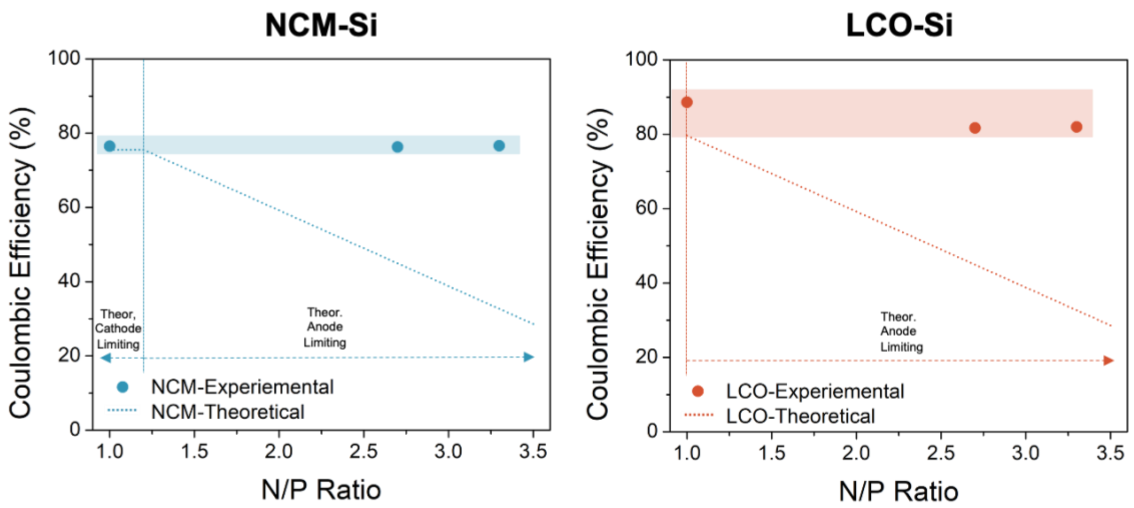

Figure S7. Theoretical and experimental Coulombic efficiency of NCM-Si and LCO Si of N/P 1 to 3.3. The circle denotes the experimental data, and the dotted line denotes the theoretical Coulombic efficiency based on the half-cell result. The colored box is to highlight the constant Coulombic efficiency achieved from the experimental data.

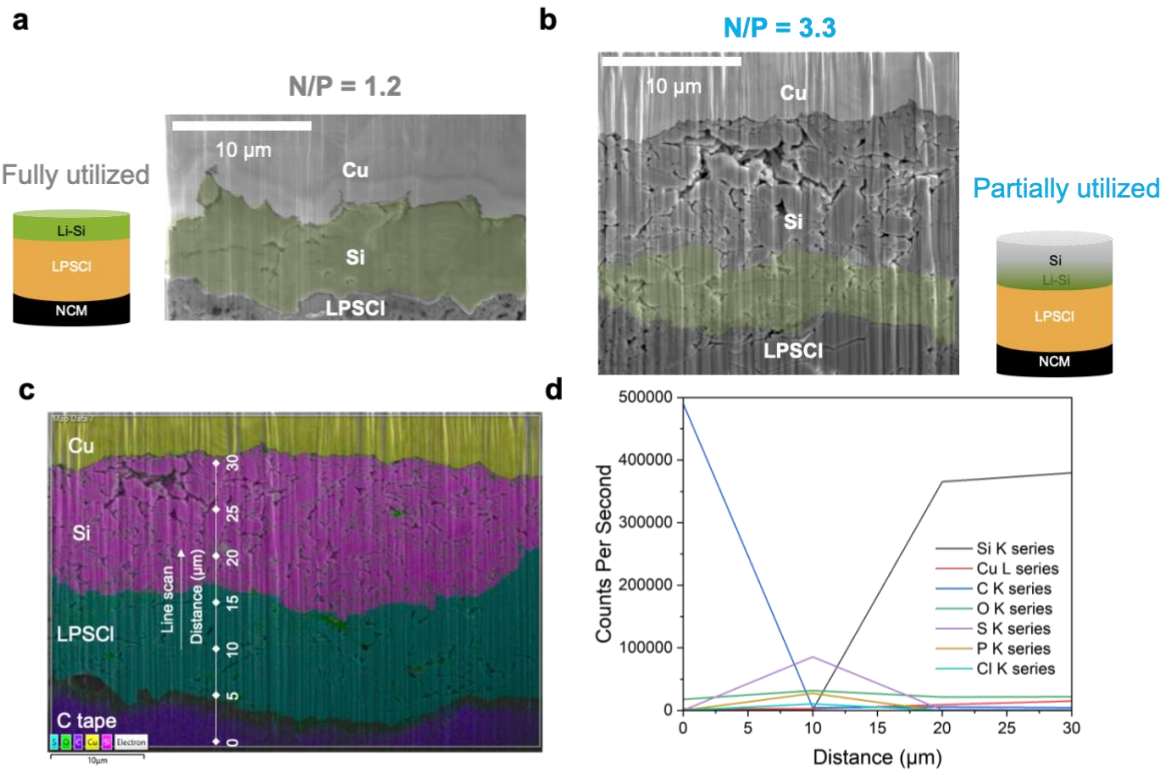

Figure S8. Morphology of Si full cell with N/P of 1.2 and 3.3. Cross-sectional FIB/SEM image of charged Si full cell of (a) N/P 1.2 and (b) 3.3. (c) EDS mapping of the charged N/P 3.3 Si cell. (d) Line scan of the charged N/P 3.3 Si cell. The line scan points and distance were denoted in Figure S8c.

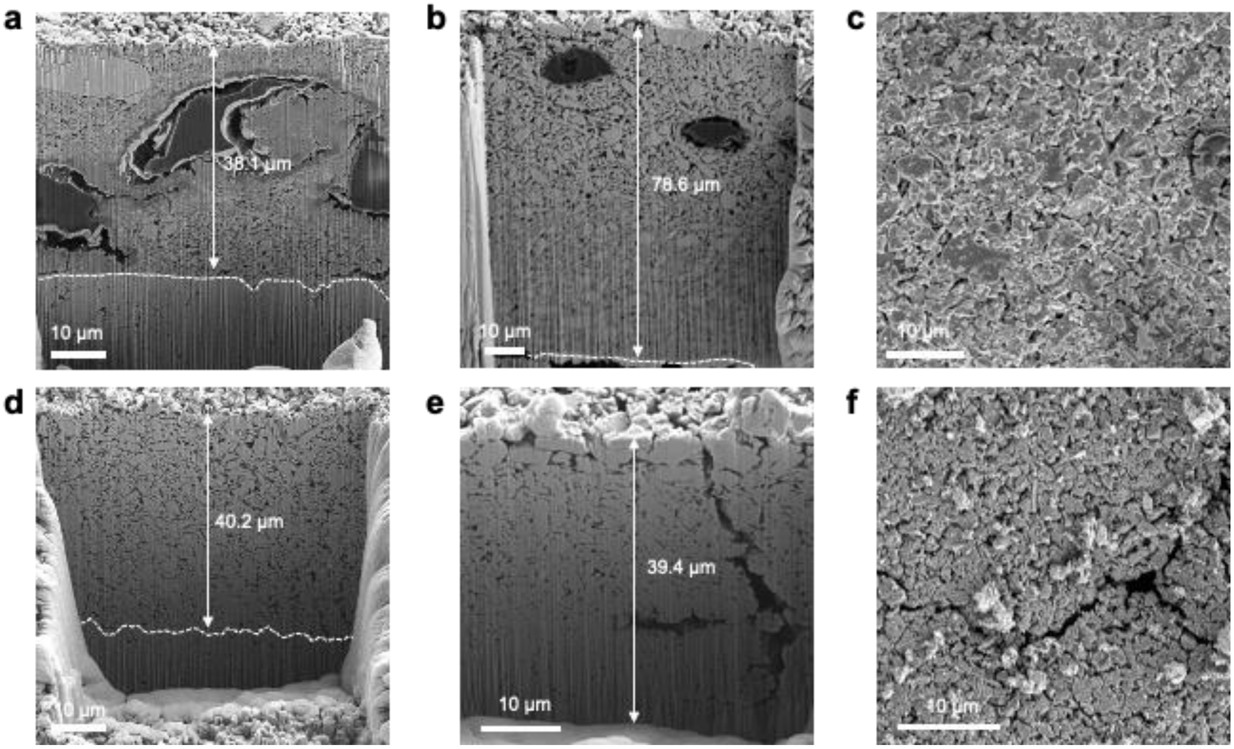

Figure S9. Morphology of Li_1Si at different state of charge. Cross-sectional FIB/SEM image of (a) pristine, (b) charged (d) discharged non-cracked spot (e) discharged cracked spot. Surface SEM image of (c) charged and (f) discharged. All images were obtained from Li_1Si samples. The charged and discharged samples were all first cycle results of Li_1Si cells.

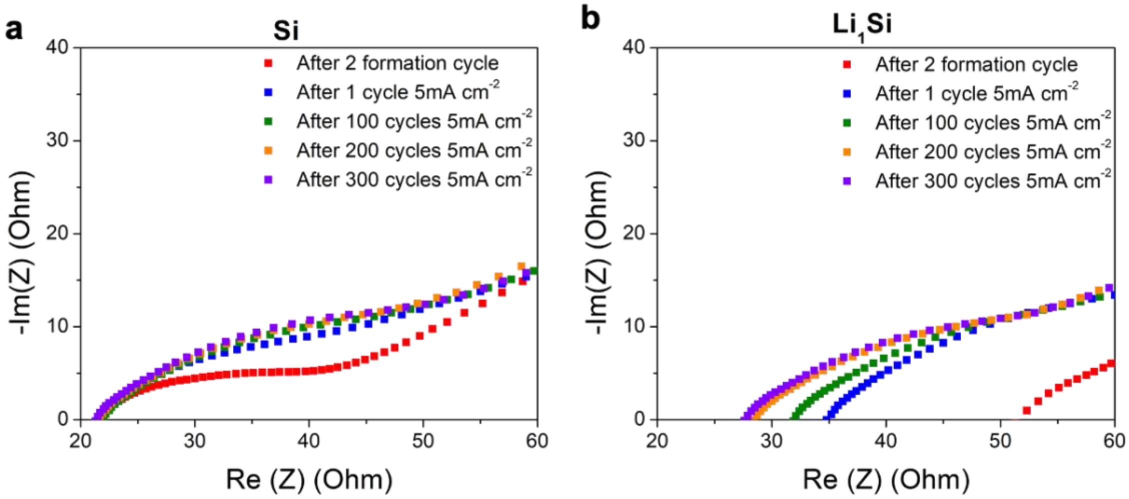

Figure S10. EIS of Si and Li_1Si upon cycling. The two cycles of formation steps at C/20 was performed prior to 5 mA cm^{-2} long cycling.

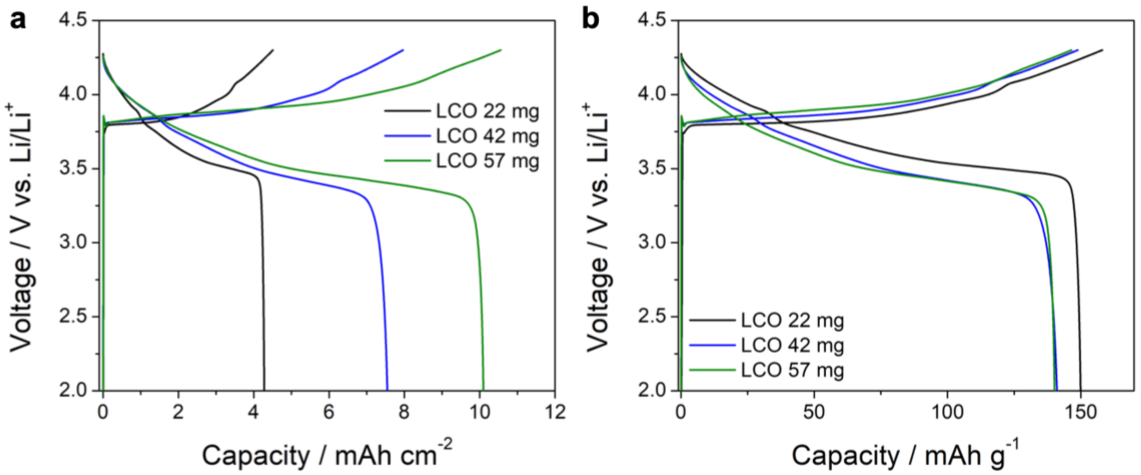

Figure S11. First cycle performance of high cathode loading Li_1Si cells. Voltage profiles of LCO cathode high loading cell paired with Li_1Si (a) areal capacity, (b) gravimetric capacity.

Supplementary References

1. Key, B. *et al.* Real-Time NMR Investigations of Structural Changes in Silicon Electrodes for Lithium-Ion Batteries. *J. Am. Chem. Soc.* **131**, 9239–9249 (2009).
